# Traumatic Brain Injury Induced Secondary Psychosis in a Young African American Male

**DOI:** 10.7759/cureus.30416

**Published:** 2022-10-18

**Authors:** Patrick D Plummer, Fahima Banu, Christian Nwabueze, Carolina D Nisenoff, Ayodeji Jolayemi

**Affiliations:** 1 Department of Psychiatry, Interfaith Medical Center, Brooklyn, USA

**Keywords:** drug-induced psychosis, schizoaffective, psychiatry and neuroscience, secondary psychosis, traumatic brain injury

## Abstract

Traumatic brain injury (TBI) is an intricate process in which the chemical balance and physical structure of the brain are altered. This medical condition’s effects range from altered mental status to an irreversible comatose state, and in severe cases even death. TBI affects millions of individuals worldwide on an annual basis. In the United States, approximately 2.87 million TBI-related emergency department (ED) visits were reported in 2014, and nearly 43% of these cases will experience long-term disabilities. These disabilities have both short- and long-term consequences on health, ranging from physical, emotional, and psychosocial changes in an individual. The goal of this case report is to highlight the morbidity of patients with TBI, with a key focus on TBI-induced secondary psychosis. While many patients recover from their symptoms of TBI within weeks to months, a subdivision of patients with TBI has permanent damage that will significantly affect the quality of their daily lives. TBI-induced secondary psychosis is the new onset of psychosis that can comprise visual, auditory, and tactile hallucinations, delusions, and disorganized thoughts. In this case report, the patient is a 22-year-old African American male who suffered a TBI at the age of 16. Prior to the patient’s TBI sustained in 2016, the patient did not have a hospital record of past psychiatric illness. In addition, the patient’s family history did not show evidence of schizophrenia, bipolar, or depression in close or distant relatives. The patient presented to the ED for a psychiatric evaluation due to psychotic behavior. In this case report, we will discuss the pathogenesis, clinical presentation, and other secondary causes of TBI-induced secondary psychosis.

## Introduction

Traumatic brain injury (TBI) is one of the leading causes of physical and mental disabilities. In the United States, more than 30% of injuries related to death involve a TBI. Each year, approximately, 64-74 million individuals worldwide sustain a TBI ranging from moderate to severe [1]. The global incidence rate of newly diagnosed TBI is estimated at 100-749 per 100,000 people, or 55.9 million individuals [1]. Males are more likely to sustain a TBI than females. The annual incidence of TBI in males is 388 per 100,000 in comparison to their female counterparts at 195 per 100,000 [1,2]. The highest rates of TBI are seen in children (0-4 years), adolescents (15-24 years), and the elderly (>65 years) population [1,2]. The causes of TBI can be falls, motor vehicle accidents, physical violence, or sports-related injuries.

Two types of injuries, primary and secondary, are involved in the pathogenesis of TBI. Primary TBI is the direct consequence of external impaction of the brain, which induces swelling, causing cerebral edema [2]. At the site of injury, the release of inflammatory cytokines contributes to vasodilation and brain edema. This new-onset edema can lead to increased intracerebral pressure and thereby the compression of the brain tissue against the skull. This results in further damage to the brain [2]. Secondary brain injuries occur within minutes to days after the initial trauma. This process consists of a chemical and inflammatory cascade that contributes to further damage. This cascade involves the depolarization of pre-synaptic neurons leading to the release of excitable neurotransmitters (NT) glutamate and aspartate. This excitable NT binds to N-methyl-D-aspartate receptors (NMDA-R), leading to an increase in intracellular calcium [2]. The influx of calcium into the cell activates enzymatic caspases and generates free radicals, which induce apoptosis in neuronal cells [2]. Depending on the severity of the injury, either type can lead to temporary or permanent brain damage. TBI causes cognitive deficits in attention, memory, information processing speed, and executive functioning [3]. Studies have shown that up to 10 years after a TBI, neuropsychological deficits can still be present. While nearly 50% of mild cases recover complete cognitive functioning, 20% of the patients require additional therapy [3].

TBI-induced psychiatric disorders include depression, bipolar, anxiety, insomnia, substance abuse, and psychosis [3]. The psychiatric symptoms following a TBI can be temporary, either confined to the first week or persistent. According to a study by Zgaljardic et al., data from a meta-analysis showed that TBI increases the incidence of psychiatric disorders including bipolar and depression [4,5]. A study conducted by Koponen et al. found that 48% of the study population developed a new axis I psychiatric disorder 30 years after their TBI. About 8% of those with new axis I psychiatric disorder developed psychotic features [6].

## Case presentation

The patient is a 22-year-old African American male with a past psychiatric history (PPHx) of schizoaffective disorder (bipolar type), and attention deficit hyperactive disorder (ADHD) diagnosed at age 4. The patient also has comorbid cocaine use disorder, cannabis use disorder, and K2 (synthetic cannabinoids) use disorder. In 2016, the patient was involved in a severe bike accident at the age of 16, where he sustained a TBI. Prior to this event, the patient had no past psychiatric history. His past medical records acquired from various medical institutions showed an extensive hospitalization history post-TBI for auditory hallucinations, suicidal ideations, and polysubstance abuse.

**Figure 1:**
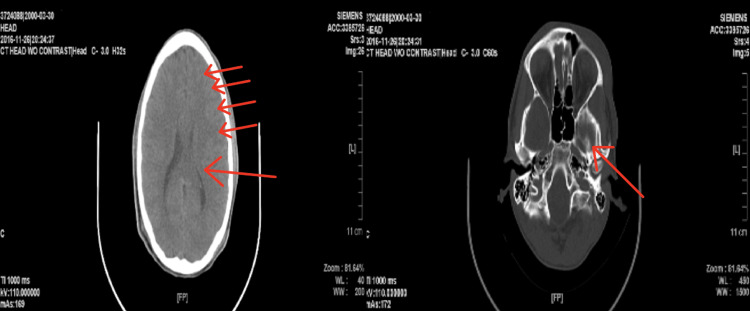
Axial CT without contrast scan of the brain was taken in 2016 (one week after the patient’s traumatic brain injury). Small red arrows show left-sided edema of the brain. A large red arrow indicates ventricular narrowing due to swelling.

**Figure 2:**
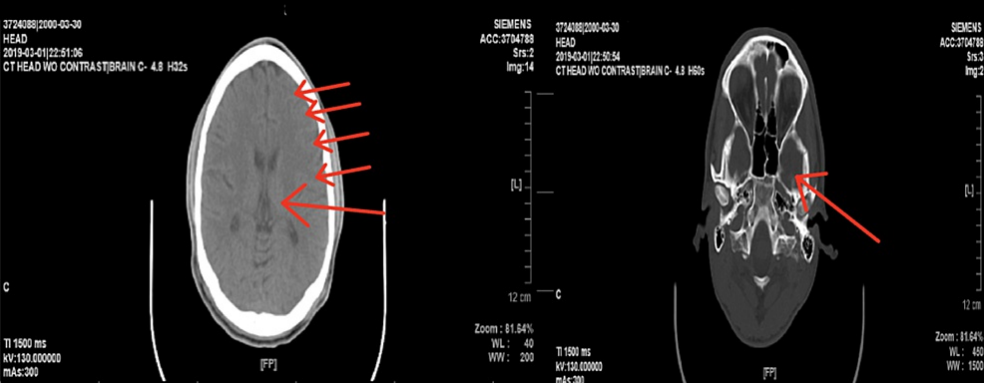
Axial CT without contrast scan of the brain was taken in 2019 (three years after the patient's traumatic brain injury). Small red arrows show no left-sided edema of the brain. A large red arrow indicates no ventricular narrowing.

In 2016, when the patient was first diagnosed with TBI, his computed tomography (CT) scan without contrast was significant for mild cerebral edema on the left side with narrowing of the lateral ventricle. The radiological report revealed no hemorrhage, white matter disease, acute fracture of the skull, ventriculomegaly, or acute sinusitis (Figure 1). In comparison, the patient’s 2019 CT without contract demonstrated no cerebral edema, infarcts, hemorrhage, white matter disease, ventriculomegaly, or fractures (Figure 2). According to the patient’s past medical records, there is no evidence of an MRI being performed near the time of the patient’s TBI.

In 2018, the patient was hospitalized for his first hypomanic episode of fast-paced thoughts, increase sexual interest and behavior, and the feeling of excessive energy, lasting for three to four days. After the patient’s first psychiatric episode in 2018, he was hospitalized for reoccurring psychiatric episodes in 2019, 2020, 2021, and 2022. The patient was diagnosed with schizoaffective disorder in 2021 when he was brought into the hospital for auditory hallucinations, hypomania, and suicidal ideations.

Based on the clinical presentation and the development of the patient’s psychosis, the patient’s TBI is likely contributing to his psychosis. After a thorough review of the patient’s past medical, family, and social history, other secondary causes of psychosis were excluded. For example, the patient had no immediate or distant family members with a psychiatric illness. According to the patient’s medical records, there were no reported past emotional or physically traumatic events before the TBI sustained in 2016. Finally, the patient increased his substance use after he developed psychosis. Therefore, the patient’s substance abuse is not the main contributory factor to his psychiatric condition.

In 2022, the patient was brought in by emergency medical services (EMS) to the psychiatric emergency department (ED) for experiencing auditory hallucinations, suicidal ideations, and disorganized thoughts. The patient was found on the street stating, “I want to hurt myself; I want to rebirth myself; I could not speak with the pope inside the church.” Inside the ED, the patient displayed agitated and paranoid behavior toward medical staff members. Due to the patient's agitated behavior, he was sedated with an intramuscular (IM) injection of 2 mg lorazepam (Ativan) and 5 mg haloperidol (Haldol). Upon re-evaluation, the patient was calm and cooperative and unable to provide adequate information on the events that led to his hospitalization. The patient states “I called emergency medical services because I don’t want anybody to take my girlfriend.” The patient admitted to smoking heroin, cigarette, and cannabis prior to his hospitalization. Table 1 displays the patient’s urine toxicology report on the day of admission. The patient was transferred to the inpatient psychiatric unit for further monitoring and rehabilitation. Once the patient was cleared by the inpatient psychiatry team, he was released back to the shelter. The patient was encouraged to follow up with his outpatient psychiatrist and continue to take his medication.

**Table 1:** The patient’s urine toxicology report on the day of admission was positive for cocaine and cannabinoid.

Component	Reference range and units	Results
Urine cocaine screen	<300 ng/mL	Positive
Urine cannabinoid screen	<50 ng/mL	Positive
Urine barbiturate screen	<200 ng/mL	Negative
Urine benzodiazepine screen	<200 ng/mL	Negative
Urine methadone	<300 ng/mL	Negative
Urine propoxyphene	<300 ng/mL	Negative
Urine ethanol	<10 ng/mL	Negative
Urine amphetamines	<1000 ng/mL	Negative
Urine opiate	<300 ng/mL	Negative
Urine phencyclidine	<25 ng/mL	Negative

In the patient's 2022 hospital admission, the patient’s schizoaffective disorder (bipolar type) was managed inpatient with 200 mg of clozapine by mouth (PO) at bedtime and lithium 600 mg 2 times a day (BID). The patient’s lithium level was measured during his most recent ED visit and determined to be < 0.3 mmol/L (reference range: 1.0-1.2). Based on the patient’s lithium level, it was determined that he was non-compliant with his outpatient medication of lithium. The patient’s lack of adherence to his medication can be contributing to his worsened psychosis and unstable mood. Upon discharge, the patient was prescribed at-home medication of 200 mg of clozapine PO daily at bedtime due to multiple recurrent episodes of psychosis with suicidal ideations. An additional medication, 30 mg mirtazapine PO daily, was added at bedtime to help stabilize the patient’s mood. The patient was counseled on the importance of adherence to his medication and frequent outpatient check-ups to monitor his leukocyte levels.

The patient's chronic history of polysubstance abuse includes cocaine, cannabis, heroin, alcohol, and tobacco. According to the patient, the frequency of his drug use increased significantly after he sustained TBI in 2016. The patient denied disclosing further information on his drug use. During the patient's hospitalization, he was counseled on his polysubstance abuse and its effect on his physical and mental health. The patient was given additional information on detoxification facilities and drug abuse support programs.

## Discussion

While secondary psychosis in patients who sustained a TBI is rare, there is significant scientific evidence that supports this diagnosis. Dopamine (DA) neurons, which are found in the midbrain, can be subdivided into their respective locations, projection sites, and behavioral functions [7]. DA neurons, from the substantia nigra project fibers to the limbic, cortical, and associative striatum [7]. Newly onset psychosis after TBI can be correlated to the secondary effects of TBI on the brain. Excessive excitability of NDMA-R via glutamate creates excessive reactive stress on the brain leading to damage in various areas [2]. The pathogenesis of schizophrenia is centered on the hyperactivity of dopaminergic neurons leading to hallucinations, delusions, and disorganized thoughts [7]. This cellular process cannot be identified by computerized image but only by a brain biopsy. Hence, in patients with TBI, it is possible for their CT or magnetic resonance imaging (MRI) to be normal, which is seen in the patient's 2019 CT scan without contrast.

In patients with TBI, physiological or emotional stress can trigger the brain to develop various psychiatric disorders. For example, a patient can develop post-traumatic stress disorder (PTSD), depression, drug abuse, and psychosis [7]. This dysregulation of the brain's chemical equilibrium leads to secondary psychosis and can present as schizophrenia, schizophreniform, or schizoaffective.

While evidence of TBI-induced psychosis is limited, a meta-analysis conducted in 2011 by Molloy et al. grouping 172 studies compared the risk of “schizophrenia” in persons with TBI versus the risk of “schizophrenia” in a control group [7]. The results from the study show that there is a significant association between TBI and schizophrenia with an odds ratio (OR) of 1.65 and a confidence interval (CI) of 1.17-2.32 [6,7]. In TBI patients, the risk of developing psychosis is significant in the second year of post-TBI recovery with an OR of 5.9 and a CI of 1.6-22.1 [7]. The third year of post-TBI recovery was also found to be a significant predictor of precipitated psychosis with an OR of 3.6 and CI of 1.0-12.3 [7]. In the case of our patient, he developed the first psychotic episode two years after the TBI and had subsequent episodes at least once every year since then. This presentation is consistent with the literature as documented above.

Other important factors contributing to the development of psychosis in younger patients (15 to 25 years of age) with newly diagnosed TBI include substance abuse, childhood trauma, and epigenetic components [8]. Routine use of cannabinoids, amphetamines, cocaine, N-methyl-D-aspartate (MNDA), and lysergic acid diethylamide (LSD) can precipitate substance-induced psychosis (SIP) [8]. Our patient in this report uses substances as indicated by the urine toxicology test was positive for both cocaine and cannabinoids. The use of cocaine and cannabis in this patient with TBI may have contributed to the development of psychosis. The differentiation between SIP and primary psychotic disorder (PPD) in young patients remains difficult to correlate [9]. The lack of psychotic features during prolonged periods of abstinence can support PPD. However, individuals predisposed to developing PPD have an increased predisposition to using substances that can unmask latent psychosis [9]. In the case of our patient, PPD is less like to precipitate the development of his psychosis. The combination of the patient's TBI and chronic substance abuse contributed to the acceleration of his psychotic episodes. Based on the case presentation, there is significant support that the patient's psychosis developed secondary to his TBI and that the chronic substance abuse accentuated his psychosis.

In a recent meta-analysis by Varesre et al., data collected from cross-sectional, case-control, and prospective cohorts on childhood trauma and the development of psychosis were found to have an OR of 2.78 [8,10]. The results from the study showed that individuals with schizophrenia have 2.72 higher odds of having traumatic events in their childhood [10]. Lastly, schizophrenia is associated with a genetic preposition. An individual with a first-degree relative with schizophrenia is at an increased risk of developing the disorder. In addition, after a thorough review of the patient’s available family history, there was no evidence of immediate or distant family members with schizophrenia, which decreases the patient’s risk of developing schizophrenia solely contingent upon genetic disposition.

## Conclusions

While TBI-induced secondary psychosis is rare, the fundamental building blocks for this condition are evident in this patient. Significant damage to the brain can disrupt its normal chemical equilibrium leading to psychosis. There is significant support in the medical literature that TBI triggers depression, insomnia, PTSD, and drug abuse. Furthermore, there is a high probability that TBI can induce other psychiatric conditions, and newly onset secondary psychosis should be considered an important differential in patients who sustained a TBI.
